# Longitudinal Dietary Intake Data in Patients with Phenylketonuria from Europe: The Impact of Age and Phenylketonuria Severity

**DOI:** 10.3390/nu16172909

**Published:** 2024-08-31

**Authors:** Alex Pinto, Kirsten Ahring, Manuela Ferreira Almeida, Catherine Ashmore, Amaya Bélanger-Quintana, Alberto Burlina, Turgay Coşkun, Anne Daly, Esther van Dam, Ali Dursun, Sharon Evans, François Feillet, Maria Giżewska, Hulya Gökmen-Özel, Mary Hickson, Yteke Hoekstra, Fatma Ilgaz, Richard Jackson, Alicja Leśniak, Christian Loro, Katarzyna Malicka, Michał Patalan, Júlio César Rocha, Serap Sivri, Iris Rodenburg, Francjan van Spronsen, Kamilla Strączek, Ayşegül Tokatli, Anita MacDonald

**Affiliations:** 1 Birmingham Children’s Hospital, Birmingham B4 6NH, UK; catherine.ashmore@nhs.net (C.A.); a.daly3@nhs.net (A.D.); evanss21@me.com (S.E.); anita.macdonald@nhs.net (A.M.); 2 School of Health Professions, Faculty of Health, University of Plymouth, Plymouth PL4 6AB, UK; mary.hickson@plymouth.ac.uk; 3 Department of PKU, Copenhagen University Hospital, 2100 København, Denmark; kirsten.ahring@regionh.dk; 4 Centro de Genética Médica, Unidade Local de Saúde de Santo António, E.P.E. (ULSSA), 4099-028 Porto, Portugal; manuela.almeida@chporto.min-saude.pt; 5 Centro de Referência na área de Doenças Hereditárias do Metabolismo, Unidade Local de Saúde de Santo António, E.P.E. (ULSSA), 4099-001 Porto, Portugal; 6 Unit for Multidisciplinary Research in Biomedicine, Abel Salazar Institute of Biomedical Sciences, University of Porto-UMIB/ICBAS/UP, 4050-313 Porto, Portugal; 7 Unidad de Enfermedades Metabólicas Congénitas, Hospital Universitario Ramón y Cajal, 28034 Madrid, Spain; amaya.belanger@salud.madrid.org; 8 Division of Inherited Metabolic Diseases, Reference Centre Expanded Newborn Screening, Department of Women’s and Children’s Health, University Hospital, 35128 Padova, Italy; alberto.burlina@unipd.it (A.B.); christian.loro@aopd.veneto.it (C.L.); 9 Department of Pediatric Metabolism and Nutrition, Faculty of Medicine, Hacettepe University, 06230 Ankara, Turkey; drturgaycoskun@gmail.com (T.C.); adursun@hacettepe.edu.tr (A.D.); ssivri@hacettepe.edu.tr (S.S.); atokatli@hacettepe.edu.tr (A.T.); 10 Division of Metabolic Diseases, Beatrix Children’s Hospital, University Medical Centre Groningen, University of Groningen, Hanzeplein 1, 9700 RB Groningen, The Netherlands; e.van.dam@umcg.nl (E.v.D.); y.hoekstra@umcg.nl (Y.H.); i.l.rodenburg@umcg.nl (I.R.); f.j.van.spronsen@umcg.nl (F.v.S.); 11 Department of Paediatrics, Reference Center for Inborn Errors of Metabolism, Hôpital d’Enfants Brabois, CHU Nancy, 54500 Vandoeuvre les Nancy, France; f.feillet@chru-nancy.fr; 12 Department of Pediatrics, Endocrinology, Diabetology, Metabolic Diseases, and Cardiology of the Developmental Age, Pomeranian Medical University, 70-204 Szczecin, Poland; maria.gizewska@gmail.com (M.G.); alicjalesniak60@gmail.com (A.L.); chmura.kasia@gmail.com (K.M.); mpatalan@hotmail.com (M.P.); kamillastraczek@wp.pl (K.S.); 13 Department of Nutrition and Dietetics, Faculty of Health Sciences, Hacettepe University, 06100 Ankara, Turkey; hgokmen@hacettepe.edu.tr (H.G.-Ö.); fatma.celik@hacettepe.edu.tr (F.I.); 14 Cancer Research UK Liverpool Cancer Trials Unit, University of Liverpool, Liverpool L69 3GL, UK; r.j.jackson@liverpool.ac.uk; 15 Nutrition and Metabolism, NOVA Medical School|Faculdade de Ciências Médicas, NMS|FCM, Universidade Nova de Lisboa, 1169-056 Lisboa, Portugal; rochajc@nms.unl.pt; 16 CINTESIS, NOVA Medical School|Faculdade de Ciências Médicas, NMS|FCM, Universidade Nova de Lisboa, 1169-056 Lisboa, Portugal; 17 Reference Centre of Inherited Metabolic Diseases, Unidade Local de Saúde, 1169-045 Lisboa, Portugal; 18 Comprehensive Health Research Centre (CHRC), NOVA Medical School|Faculdade de Ciências Médicas, NMS|FCM, Universidade Nova de Lisboa, 1169-056 Lisboa, Portugal

**Keywords:** phenylketonuria, natural protein intake, protein equivalent from protein substitute intake, total protein intake, phenylalanine intake

## Abstract

In phenylketonuria (PKU), natural protein intake is thought to increase with age, particularly during childhood and adolescence. Longitudinal dietary intake data are scarce and lifelong phenylalanine tolerance remains unknown. Nine centres managing PKU in Europe and Turkey participated in a retrospective study. Data were collected from dietetic records between 2012 and 2018 on phenylalanine (Phe), natural protein, and protein substitute intake. A total of 1323 patients (age range: 1–57 y; 51% male) participated. Dietary intake data were available on 1163 (88%) patients. Patient numbers ranged from 59 to 320 in each centre. A total of 625 (47%) had classical PKU (cPKU), *n* = 357 (27%) had mild PKU (mPKU), *n* = 325 (25%) had hyperphenylalaninemia (HPA), and *n* = 16 (1%) were unknown. The mean percentage of blood Phe levels within target ranged from 65 ± 54% to 88 ± 49%. When intake was expressed as g/day, the mean Phe/natural protein and protein equivalent from protein substitute gradually increased during childhood, reaching a peak in adolescence, and then remained consistent during adulthood. When intake was expressed per kg body weight (g/kg/day), there was a decline in Phe/natural protein, protein equivalent from protein substitute, and total protein with increasing age. Overall, the mean daily intake (kg/day) was as follows: Phe, 904 mg ± 761 (22 ± 23 mg/kg/day), natural protein 19 g ± 16 (0.5 g/kg/day ± 0.5), protein equivalent from protein substitute 39 g ± 22 (1.1 g/kg/day ± 0.6), and total protein 59 g ± 21 (1.7 g/kg/day ± 0.6). Natural protein tolerance was similar between males and females. Patients with mPKU tolerated around 50% less Phe/natural protein than HPA, but 50% more than cPKU. Higher intakes of natural protein were observed in Southern Europe, with a higher prevalence of HPA and mPKU compared with patients from Northern European centres. Natural protein intake doubled with sapropterin usage. In sapropterin-responsive patients, 31% no longer used protein substitutes. Close monitoring and optimisation of protein intake prescriptions are needed, along with future guidelines specifically for different age groups and severities.

## 1. Introduction

Phenylketonuria (PKU) is an inherited metabolic disorder of phenylalanine (Phe) metabolism. The gene producing phenylalanine hydroxylase (PAH) fails to be expressed, leading to impaired Phe metabolism and its conversion to tyrosine. PAH activity also requires the pterin co-substrate tetrahydrobiopterin (BH4) for activity [1]. PKU is a neurological disorder, with Phe accumulating in the brain tissue and biological fluids and leading to neurocognitive and neuropsychiatric sequelae, if untreated. In most countries, it is diagnosed by newborn screening and is treated when blood Phe levels are consistently >360 µmol/L [2].

The degree of PAH loss of function determines the PKU phenotype and correlates with the severity of the disorder [3]. Classical PKU (cPKU) is a result of complete or near-complete deficiency of PAH activity and is the most frequent type of PKU globally [4]. The most common variants of PAH in the BIOPKU database [5] are c.1222C>T (p.Arg408Trp) and c.1066-11G>A (p.Gln355_Tyr356insGlyLeuGln); these are severe variants that essentially abolish PAH activity. Other variants with varying effects on the activity of PAH may be classified as mild PKU (mPKU) and hyperphenylalaninemia (HPA) associated with residual enzyme activity, and thereby a higher enzyme activity [5]. The severity of PAH deficiency is increasingly analysed by the genotype predictive value [3]. If the diagnostic Phe levels prior to treatment commencement is used, then HPA is defined by a pre-treatment blood Phe level of 361–600 μmol/L, mPKU > 600–1200 μmol/L, and cPKU > 1200 μmol/L [3].

The traditional treatment of PKU is a Phe-restricted diet, supplemented with a Phe-free/low Phe protein substitute, special low protein foods (SLPFs), foods low in protein (containing protein ≤ 0.5 g/100 g), and fruits and vegetables containing Phe ≤ 75 mg/100 g [6]. The treatment aim in Europe is to maintain a blood Phe between 120 and 360 µmol/L in children up to the age of 12 years, and between 120 and 600 µmol/L aged ≥12 years [2]. In the USA, it is recommended that blood Phe should be ≤360 µmol/L throughout life [7], this is still above the physiological range (<120 µmol/L) [8]. In PKU, although normal growth and nutritional status can be attained, clinical problems such as inattention, poor memory and executive function deficits, anxiety, and social and emotional problems commonly occur [9].

Dietary Phe is an indispensable amino acid, and some is essential for protein synthesis, tissue growth, and maintenance requirements [10,11,12]. In PKU, this is referred to as the “natural Phe/protein tolerance” and the amount of Phe allocated is individually determined for each patient [13,14]. Phe tolerance is defined as the maximum amount of Phe/natural protein intake that will maintain blood Phe levels within the therapeutic target range [13]. It is important that the maximum Phe tolerance is attained so that the iatrogenic consequence of overtreatment is avoided [2]. Phe tolerance is pragmatically decided by titrating Phe/natural protein intake until blood Phe levels are consistently maintained within the target range. Generally, if blood Phe levels are above or below the target range, the Phe intake is lowered or increased accordingly. In newly diagnosed infants with PKU, it can take time to establish the maximum Phe tolerance, and may even remain underestimated, particularly if blood Phe consistently remains within target and a challenge with extra Phe intake is not considered [15]. Phe tolerance is commonly associated with the severity of PKU, with studies commonly describing patients with cPKU with an overall tolerance of ≤500 mg/day of Phe (equivalent to ≤10 g/d of natural protein) at various ages during life [2]. In addition to the severity of PKU, factors such as inadequate energy intake or illness can cause catabolism, increasing blood Phe levels, and can negatively impact on the amount of natural protein tolerated, while extra protein substitute may enhance Phe tolerance to some extent [16].

Dietary Phe tolerance is expected to improve in a subset of individuals with PAH deficiency in response to treatment with oral sapropterin dihydrochloride, a synthetic form of the natural BH4 cofactor [17,18]. These individuals have PAH genotypes associated with some residual PAH enzyme activity, and patients with cPKU are excluded. Sapropterin is considered to have a chaperone function that stabilises and activates the variant PAH protein [19,20]. Only 20–40% of PAH-deficient patients are sapropterin-responsive [21,22,23], and, in most, partial dietary Phe restriction is still necessary [24], although long-term Phe tolerance is rarely described.

An alternative pharmaceutical treatment for patients > 16 year of age with PKU is Pegvaliase [25]. This enzyme substitution therapy recombinantly produces PEGylated Phe ammonia lyase (PAL) [26]. It is derived from *cyanobacterium Anabaena variabilis* and converts circulating Phe to trans-cinnamic acid and clinically non relevant amounts of ammonia [27]. It lowers blood Phe and enables many patients to have an unrestricted diet while maintaining blood Phe levels in the target therapeutic range or even at physiological levels. However, this treatment is administered by daily subcutaneous injection and is associated with immune-mediated hypersensitivity reactions [28]. The number of countries with access to Pegvaliase is still very limited, while the global numbers of patients benefiting from this treatment is unknown [29,30].

Very little has been published about the changes in lifelong dietary Phe tolerance in PKU, particularly across the different severities [13]. Although studies commonly report the Phe/natural protein intake as part of their secondary outcomes, most studies are cross-sectional, provide limited dietary intake data, and often describe childhood Phe intake only [13]. Due to insufficiency of evidence in individual studies, a recent systematic review and meta-analysis provided a better understanding of lifelong Phe/natural tolerance [13]. Data from 37 articles were analysed, including 2464 patients with PKU. A meta-analysis of 18 studies showed that Phe and natural protein tolerance slightly increased from birth until 10 years with an average Phe intake of <500 mg/day (range: 267–460) and natural protein intake of <13 g/day (range: 5–13). With the onset of pubertal growth spurt, there was an additional increase in Phe tolerance of +250 mg/day (+5 g/day of natural protein), allowing a Phe intake of ≈710 mg/day (18 g/day of natural protein) [13]. At the end of adolescence, natural protein tolerance reached its highest peak, with an average daily tolerance of 32 g/day, remaining consistent through adulthood (1160–1810 mg/day Phe). However, historically, variable target blood Phe levels were used by different centres worldwide, small study sample sizes (particularly for adults), incomplete and diverse data on PKU severity, and inadequate or unclear reporting of dietary Phe/natural protein intake complicated the results, and this reinforced the need to carefully interpret the results [13].

Accurately estimating Phe/natural protein tolerance in PKU is challenging [31]. Different PKU centres use dissimilar protocols for controlling the amount of daily Phe/natural protein consumed. Some calculate all the Phe/natural protein intake from all foods, whereas others permit the unlimited use of fruit and vegetables (except potatoes) containing Phe ≤ 75 mg/100 g as it does not have a negative impact on blood Phe control [32,33,34]. This may partly explain the heterogeneity between study results investigating the dietary intakes. Additionally, foods containing protein ≤ 0.5 g/100 g are allowed without measurement in certain countries. Some countries may use an exchange system for allocating Phe intake and it has been shown that different dietary systems for allocating Phe achieve similar blood Phe control [35]. In countries that recommend an exchange system, variable definitions to define one exchange are used (e.g., 20 mg, 25 mg, 50 mg Phe), and there is a disparity between the amount of Phe that is prescribed compared with the actual amount eaten. In a recent study from the UK, using a 1 g protein exchange system (equivalent to around 50 mg Phe), patients consumed 40% more natural protein (+4 g/day) than prescribed without deterioration in blood Phe control [36]. In addition, a higher recommended blood Phe target range may permit more dietary Phe/natural protein. Overall, there are no studies assessing lifetime Phe/natural protein tolerance in patients with PKU. Similarly, no studies account for the different variable factors influencing Phe/natural protein tolerance. To date, only one systematic review (including studies mostly from Europe, *n* = 30/37 studies) has described the changes in Phe/natural protein tolerance throughout a lifetime, but there were a number of limitations (e.g., different ways of presenting dietary intake analysis, and different target levels used to define good metabolic control) [13]. Our aim was to assess dietary intake in a large cohort of patients with PKU from Europe and Turkey.

## 2. Materials and Methods

### 2.1. Participating Centres

Nine PKU centres from Europe and Turkey participated in this study. Dietitians and clinicians who were part of the European Nutritionist Expert Panel on PKU (ENEP) participated from Centres A (Ankara, Turkey), B (Birmingham, UK), C (Groningen, The Netherlands), E (Porto, Portugal), G (Copenhagen, Denmark), H (Madrid, Spain), and I (Padova, Italy). Two other centres also participated: Centre D (Nancy, France) and F (Szczecin, Poland). All except one paediatric centre (UK) were caring for both paediatric and adult patients. There were *n* = 2 centres from North Europe, *n* = 2 from central Europe, *n* = 1 from Eastern Europe, *n* = 3 from Southern Europe, and *n* = 1 from Turkey.

### 2.2. Patient Selection

Patients with PKU were included if they were diagnosed by (1) newborn screening and (2) early (by age 3 months) and continuously treated with a Phe-restricted diet and/or pharmacological treatments. Exclusion criteria were (1) late diagnosis (aged >3 months), (2) delayed initiation of treatment (aged > 3 months), (3) a co-existing condition that may have a negative impact on metabolic control (e.g., leukaemia), and (4) pregnancy (Phe tolerance is expected to increase after the second and third trimester of pregnancy).

### 2.3. Study Design and Data Collection

This paper reports the data on protein and Phe intakes from a retrospective longitudinal study assessing the metabolic control of patients with PKU, with data collected during the period 2012 to 2018. Study design and overall data collected were reported in detail by Pinto et al., [35] in their study “Blood phenylalanine levels in patients with phenylketonuria from Europe between 2012 to 2018: is it a changing landscape?”

In this paper, we describe the prescribed intakes of natural protein and Phe, total protein, and protein equivalent intake from protein substitutes. Type of protein substitutes used (L-amino acids [L-AA]), glycomacropeptide [GMP], and large neutral amino acids [LNAA]) were also collected. In addition, demographic data, PKU variants (when data were available, except for Ankara, as their ethical permission excluded variants), diagnostic blood Phe levels, blood Phe levels during follow-up, use and date of starting sapropterin, body weight, system for allocating dietary Phe (e.g., calculation of all Phe vs. exchange system), and number of dedicated dietitians caring for PKU in each centre were obtained.

Three centres (Centres A, D, and F) collected their own data, and in the remaining centres, data were collected by A.P. from dietetic and medical records.

### 2.4. Statistical Analysis

The aim was to recruit all eligible patients being treated and followed up by each centre; hence, a sample size calculation was not performed. Natural protein, Phe, protein equivalent from protein substitute, and total protein intakes were the primary outcomes of this study. Statistical significance was defined by *p*-value < 0.05 and reported within 95% confidence intervals. Data were summarized as mean ± SD or median (range). The statistical analysis was performed by R.J. using software R (version 3, R Foundation for Statistical Computing, Vienna, Austria).

### 2.5. Ethical Aspects

The Declaration of Helsinki (52nd WMA General Assembly, Edinburgh, UK, October 2000) and Good Clinical Practice guidelines were followed during the study. Each centre’s ethics committee approved the project individually.

## 3. Results

### 3.1. Treatment Centres’ Characteristics

Data were collected on 1323 patients with PKU from *n* = 9 centres in Europe and Turkey. Dietary intake data were available from 1163 patients with PKU (most of the missing data were from patients with HPA who were given an unrestricted/normal diet). The principles of the dietary systems used to calculate daily Phe/protein intakes by all the participating centres are given in detail in Table 1. Six centres used a protein/Phe exchange system to allocate natural protein. Only four centres permitted fruit and vegetables containing Phe ≤ 75 mg/100 g (except potatoes) in unlimited amounts without calculation of their Phe content (Centres B, C, E, and H). Various sources of protein substitute were prescribed, including amino acid-based protein substitutes (L-AAs), glycomacropeptide-based protein substitute (GMP), and large neutral amino acids (LNAAs). When data on the protein substitute source (*n* = 723) were available, at the final annual assessment, the protein substitutes prescribed were L-AA, *n* = 621; GMP only, *n* = 22; GMP + L-AA, *n* = 19; GMP + LNAA, *n* = 5; GMP + L-AA + LNAA, *n* = 1; LNAA only, *n* = 30; LNAA + L-AA, *n* = 25.

Protein substitutes were reimbursed in all the centres either by the national health systems or insurance, but this did not apply to special low protein foods (SLPFs). Although provided by the state/national health system in Centres B, D, E, G, H, and I, SLPFs were not reimbursed in Centre C (Groningen, The Netherlands) and Centre F (Szczecin, Poland), and only partially reimbursed in Centre A (Ankara, Turkey). In Centre C (Groningen, The Netherlands), a tax reduction system was applied (dependent on family income) to cover the costs of SLPFs if natural protein tolerance was <20 g/day.

During the study period, 222 (17%) patients were prescribed sapropterin. None of the patients were given Pegvaliase. Sapropterin was available in most centres, except for Centre F (Szczecin, Poland) and B (Birmingham, UK—research access only). LNAAs were given as a treatment option in adults in Centres A, E, G, and I for patients aged 16 and over. Dietary intake was included from 61 patients on LNAA.

The percentage of patients with dietary intake data ranged from 56% to 100% in each centre, with an overall mean of 90%. Dietary data were not available in all patients, especially in HPA patients. Target blood Phe levels are given in Table 1.

### 3.2. Subjects Characteristics

The characteristics of the study sample were described in detail elsewhere [35]. The number of patients ranged from 59 to 320 in each centre. The patient cohort’s median age during the data collection period was 16 years (range: 1 to 57 years). Two-thirds (67%) of the patient cohort were children or adolescents (3% infants < 12 m, *n* = 46; 43% mid-childhood aged 1–12 y, *n* = 567, 20% adolescents aged 13–19 y, *n* = 268, and 33% adults, *n* = 442).

The number of HPA, mPKU, and cPKU per centre is presented in Supplementary Table S1.

In total, 625 patients (47%) were classified with cPKU, 357 with mPKU (27%), 325 with HPA (25%), and 16 (1%) were unknown. In four of nine centres (centres A, C, E, and I), more than half of their patient cohort had a milder type of PKU (mPKU or HPA). In the remaining five centres, the most common phenotype was cPKU, ranging from 52 to 76% in each patient cohort. Centre I had the highest HPA population (51%), while Centre B had no patients with HPA. Most centres with HPA did not have dietary data available, except Centre E. The highest number of mPKU was in Centre E (48%).

### 3.3. Overall Data on Metabolic Control and Prescribed Dietary Protein/Phe Intakes Per Centre

Overall metabolic control and dietary intake data for each centre are presented in Table 2. Most centres had more than 70% of blood Phe levels within the therapeutic target range, with the mean percentage ranging from 65% in Centre G to 88% in Centre I.

Data on dietary intakes were available on *n* = 1163 (88%) patients from nine centres (Table 2).

#### 3.3.1. Phenylalanine and Natural Protein Intakes Per Centre

Overall, daily mean dietary Phe and natural protein intakes were 904 ± 761 mg/day and 19 ± 16 g/day, respectively. When data were expressed as per kg body weight, mean Phe intake was 22 ± 23 mg/kg/day and mean natural protein intake was 0.5 ± 0.5 g/kg/day.

The highest and lowest mean daily Phe and natural protein prescriptions were found in Centre E (2097 mg/day; 45 g/day; 1.0 g/kg/day) and Centre F (379 mg/day; 8 g/day; 0.3 g/kg/day), respectively.

#### 3.3.2. Protein Equivalent Intake from Protein Substitutes Per Centre

The patients were prescribed a mean dose of 39 ± 22 g/day protein equivalent from protein substitutes. The actual intake per kg body weight was 1.1 ± 0.6 g/kg.

Mean protein equivalent intake from protein substitute ranged from 17 g/day in Centre A (0.7 g/kg/day) to 52–53 g/day in Centres B (2 g/kg/day) and H (1.2 g/kg).

#### 3.3.3. Total Protein Intakes Per Centre

Total daily protein intake including natural protein and protein intake from protein substitute was 59 ± 21 g/day, corresponding to 1.7 ± 0.6 g/kg/day intake.

The total protein prescription was lowest in Centre A (36 g/day; 1.3 g/kg/day). Mean total protein prescription per day was the highest in Centre E (80 g/day) and B in g/kg/day (2.3 ± 0.7 g/kg/day).

### 3.4. Dietary Prescribed Protein/Phenylalanine Intakes by Gender and Age

Table 3 shows the data on mean daily intakes by gender and age groups.

#### 3.4.1. Phenylalanine and Natural Protein Intakes by Gender and Age Group

Mean Phe intake was similar between males (1027 ± 1200 mg/day; *n* = 657) and females (881 ± 875 mg/day; *n* = 666), *p* = 0.218. Mean natural protein intake was 22 ± 24 g/day (0.5 ± 0.6 g/kg/day) in males and 19 ± 19 g/day (0.5 ± 0.6 g/kg/day) in females, *p* = 0.25.

Analysis by age group indicated that mean Phe and natural protein intakes increased from birth (318 mg/day; 7 g/day) until the beginning of adolescence (750 mg/day; 16 g/day). During adolescence, there was >500 mg/day increase in Phe intake, corresponding to a >10 g/day increase in natural protein intake compared with the school-age period. This nearly doubled during pre-school and school-age and quadrupled in adolescence compared with the first year of life. After adolescence, Phe/natural protein intake remained stable (19–30 years), and even slightly decreased >30 years of age.

When data were expressed as intake per kg body weight, the maximum Phe/natural protein intake was observed in the first year of life (34 mg/kg/day and 1.0 g/kg/day) and decreased in pre-school (29 mg/kg/day and 0.8 g/kg/day) and school-age children (22 mg/kg/day and 0.5 g/kg/day). There was no further decrease until the end of adolescence. In adults, it was lower than in children and adolescents.

#### 3.4.2. Protein Equivalent Intake from Protein Substitutes by Gender and Age Group

There was no difference in mean protein equivalent intake from protein substitute in males (35 ± 25 g/day; 1.0 ± 0.7 g/kg/day) and females (35 ± 26 g/day; 1.0 ± 0.7 g/kg/day), *p* = 0.83.

Similar to the change in Phe/natural tolerance, protein equivalent from protein substitute prescriptions were higher in the early years of childhood until the end of adolescence. The mean dose of protein equivalent intake (per kg body weight) from protein substitute was 1.1 g/kg/day in infants, approximately 1.2 g/kg/day in pre-school and school-age children, 0.8 g/kg/day in adolescents, and 0.6 g/kg/day in adults.

#### 3.4.3. Total Protein Intakes by Gender and Age Group

The mean total protein intake was similar between males and females (60 ± 28 g/day; 1.8 ± 0.7 g/kg/day and 57 ± 25 g/day; 1.6 ± 0.7 g/kg/day, respectively), *p* = 0.85. The analysis for age groups showed that total protein intakes gradually increased with age from a mean intake of 17 g/day (2.4 g/kg/day) in the first year of life to 75 g/day (1.3 g/kg/day) in adolescence. In adults, total protein intakes remained at 78–80 g/day and 1.0–1.2 g/kg/day.

Overall, the total protein intakes met the safe level of protein intake (WHO/FAO/UNU 2017) for age. Mean total protein intakes (per kg of body weight) in infants and children were around 2 times higher compared with the safe levels of protein intake for age (WHO/FAO/UNU 2017), whilst the adolescents consumed 1.5 times and adults 1.2–1.4 times more than the recommended safe amounts. There were 50 patients (4%) who had lower levels of total protein intake (in g/kg/day) compared with the safe levels of protein intake. Twenty-nine (58%) were cPKU, eighteen (36%) mPKU, and three (6%) HPA. Twenty-six (52%) were from Centre A, 9 (18%) from Centre C, 7 (14%) from Centre G, 5f (10%) from Centre D, 2 (4%) from centre I, and 1 (2%) from centre E. One patient (2%) was aged 2–5 y, 2 (4%) aged 6–12 y, 15 (30%) aged 13–18 y, 25 (50%) aged 19–30 y, and 7 (14%) aged > 30 y. In some patients, low total protein intakes were associated with overweight, as protein intake was calculated using actual patient weight and not their ideal body weight. Therefore, 24 of 50 patients (48%) were overweight with a total protein intake less than safe levels of protein intake.

### 3.5. Dietary Prescribed Protein/Phenylalanine Intakes by PKU Severity

Table 4 summarizes the dietary protein/Phe intakes of patients in each age group analysed by severity of PKU, and Table 5 describes the mean differences in dietary protein/Phe intakes between PKU severities.

#### 3.5.1. Phenylalanine and Natural Protein Intakes by PKU Severity

During infancy, mean Phe and natural protein tolerance of mPKU and cPKU were similar. However, they were approximately 2 to 3 times higher in infants with HPA (*p* < 0.001).

In children and adolescents, compared with patients with cPKU, Phe/natural tolerance was >2 times and >4 times higher in mPKU and HPA, respectively.

The highest Phe/protein tolerance during adulthood was observed in young adults (19–30 years) with HPA (2851 mg/day, 24 mg/kg/day), which was 1.5 to 3 times higher than mPKU and cPKU patients in the same age group.

Overall, in all age groups, cPKU had a lower Phe/natural protein tolerance compared with mPKU (*p* < 0.001) and HPA patients (*p* < 0.001).

Mean overall Phe intake was higher in patients with HPA compared with mPKU (2270 vs. 1262 mg/day and 29 vs. 28 mg/kg/day, *p* < 0.001). Natural protein intake was also higher in HPA compared with mPKU (48 vs. 26 g/day and 1.3 vs. 0.7 g/kg/day, *p* < 0.001).

When compared with cPKU, mean overall Phe intake was higher in mPKU (1262 vs. 593 mg/day and 28 vs. 16 mg/kg/day, *p* < 0.001). Similarly, natural protein intake was higher in patients with mPKU compared with cPKU (26 vs. 13 g/day and 0.7 vs. 0.4 g/kg/day, *p* < 0.001).

#### 3.5.2. Protein Equivalent Intake from Protein Substitutes by PKU Severity

Infants with cPKU were prescribed a mean protein equivalent intake of 12 g/day (1.8 g/kg/day) from protein substitute which was >1.5 times higher than in infants with mPKU (*p* < 0.001). Protein substitute intake of infants with HPA was minimal (1 g/day; 0.1 g/kg/day).

In children and adolescents with HPA, protein equivalent from protein substitute prescriptions were markedly lower than in mPKU (*p* < 0.001) and cPKU (*p* < 0.001) and reached a maximum amount of 13 g/day at the end of adolescence. Protein substitute prescriptions in children and adolescents with cPKU were approximately 1.3 to 1.5 times higher than in mPKU (*p* < 0.001).

Adults with cPKU had a mean intake of protein equivalent of 50 g/day from protein substitutes, which was around 1.2 times higher than in mPKU (approximately 40 g/day). Although younger adults (19–30 years) with HPA had a lower protein substitute prescription (1.3–1.8 times less) than mPKU and cPKU in the same age group, the intakes were similar after the age of 30 years. Overall mean protein equivalent from protein substitutes intake was higher in patients with cPKU than mPKU (44 vs. 32 g/day and 1.2 vs. 0.9 mg/kg/day, *p* < 0.001).

Overall, protein substitute intake by HPA patients was minimal.

#### 3.5.3. Total Protein Intakes by PKU Severity

Total protein intakes in g/day of all PKU forms were similar in each age group (cPKU vs. HPA, *p* = 0.964 and cPKU vs. mPKU, *p* = 0.272). However, when expressed in grams per kg body weight, cPKU and mPKU infants consumed approximately twice the amount of total protein intake of infants with HPA (1.4 g/kg/day vs. 2.6 g/kg/day). Although there were small differences in total protein intake per kg body weight during childhood and adolescence between the three phenotypes, no further difference was observed in adulthood.

Mean overall daily total protein intake was similar in mPKU compared with HPA (61 vs. 60 g/day, *p* = 0.964) but significantly higher in mPKU when expressed as mg/kg/day (1.5 vs. 1.7 mg/kg/day, *p* = 0.044). Mean overall total protein intake was similar in cPKU compared with mPKU (60 vs. 57 g/day, *p* = 0.272; 1.7 vs. 1.7 g/kg/day, *p* = 0.788).

### 3.6. Dietary Prescribed Phenylalanine/Natural Protein Intakes Comparing Patients on Sapropterin vs. Diet-Only Treatment

Table 6 presents dietary intake data on Phe, natural protein, protein equivalent from protein substitute, and total protein intake in patients taking sapropterin compared with patients on dietary only treatment. Patients treated with sapropterin (*n* = 222) had a mean dose of sapropterin of 15 ± 5 mg/kg/day and a mean age of 15 ± 9 y during the study period.

The mean Phe and natural protein intake of PKU patients using sapropterin (1649 mg/day, 35 g/day, and 0.9 g/kg/day) were approximately twice the amount tolerated by diet-only treated patients (846 mg/day, 19 g/day, and 0.5 g/kg/day, *p* < 0.001).

Patients on dietary treatment alone were prescribed 1.5 to 2 times more protein substitute compared with patients prescribed sapropterin (37 g/day, 1.2 g/kg/day vs. 24 g/day, 0.6 g/kg/day, *p* < 0.001). Sixty-nine patients (31%) taking sapropterin did not require a protein substitute.

However, total protein intakes were similar between the two groups (BH4 vs. diet-only: 63 g/day and 1.5 g/kg/day vs. 59 g/day and 1.7 g/kg/day, *p* = 0.067).

## 4. Discussion

This was an observational, longitudinal and retrospective study in a large patient cohort (*n* = 1323) from eight European centres and one Turkish centre with PKU describing intakes of Phe, natural protein, and protein equivalent from protein substitutes in different age groups, gender, and PKU severity. Although in PKU, a Phe-restricted diet has been prescribed for over seven decades, little is known about Phe tolerance throughout life and many studies only report dietary intake data as their secondary outcome. When expressed as mg/day and g/day, the mean Phe, natural, and protein equivalent intake from protein substitute gradually increased with age, reaching a peak in adolescence (between 13 and 18 years of age), and then remained consistent during adulthood. When intake data were expressed as per kg body weight (g/kg/day), there was a decline in Phe/natural protein, protein equivalent from protein substitute, and total protein with increasing age. Natural protein tolerance was comparable between males and females. Patients with mPKU tolerated around 50% less Phe/natural protein compared with HPA, and cPKU patients only tolerated 50% of the Phe/natural protein intake of mPKU patients. Higher intakes of natural protein were observed in Southern Europe, with a higher prevalence of HPA and mPKU compared with patients from Northern European centres. Natural protein intake doubled with sapropterin usage in BH4-responsive patients, with 31% no longer using protein substitutes.

It is well established that patients with cPKU tolerate ≤500 mg/day of Phe (≤10 g/day), which is usually derived from fruit, vegetable, and cereal sources, but little information has been published about the Phe tolerance of patients with mPKU and HPA [37]. In our study, we found that mean lifetime natural protein intake was 48 g/day in HPA and 26 g/day in mPKU compared with a mean intake of 13 g/day in cPKU. The difference in Phe/natural protein tolerance between the classical and milder forms showed a similar trend in all age groups, such that the tolerance in mPKU and HPA patients was approximately 1.5 up to 4 times higher than in cPKU.

Age was another factor affecting Phe/natural tolerance. In this longitudinal study, daily Phe/natural protein intake increased with age, similar to what was described in a recent systematic review and meta-analysis [13]. However, the lack of data on Phe/natural protein tolerance in patients > 40 years of age was one of the limitations of the meta-analysis. Our data for cPKU were comparable to van Spronsen et al. [38], who described patients tolerating between 35 and 45 mg/kg of Phe in the first year of life (a mean of 38 mg/kg/day in our study), decreasing to 14 mg/kg at the age of 10 y (also a mean of 14 mg/kg in our study). We further provided data on Phe tolerance in a relatively large sample of adults (*n* = 442), which varied between 7 and 18 mg/kg/day.

These study findings assist in patient guidance about expected natural protein tolerance at different life stages and help manage their expectations. Attaining the maximum natural protein tolerance is essential to optimize growth and body composition, and aid dietary adherence, ultimately easing the burden of dietary care and costs of overtreatment. It also enables a personalized approach to dietary care. In clinical practice, the same dietary method is commonly applied to all patients irrespective of PKU severity, but, potentially, a more flexible approach can be adopted for mPKU. In a cohort of 99 patients with PKU, Gomes et al. [39] showed that patients with mPKU were able to eat more and varied animal protein sources and had a higher natural protein/protein equivalent from protein substitute ratio in comparison with cPKU. Patients with mPKU should be able to eat a wider range of foods, possibly without calculation of Phe/natural protein, particularly from fruits and vegetables, whose Phe content is more than 75 mg/100 g. Caregivers of patients with mPKU could be encouraged to eat higher biological quality protein food sources such as milk, cheese, yoghurt, and eggs from weaning age. This will help increase the dietary quality and variety, and potentially help reduce longer term food neophobia and aversive eating patterns in some patients, which are issues commonly reported in patients with PKU [40]. It is also expected that patients with mPKU should be able to tolerate GMP-based protein substitutes without calculation of their Phe content.

Understanding the expected Phe tolerance of patients, particularly with HPA and mPKU, may avoid overtreatment. Over restriction of natural protein is a potential concern in PKU, with Phe tolerance potentially remaining unchallenged if blood Phe remains comfortably within target range. Studies from Centre E demonstrated that 65% of the patients, mainly with HPA or mPKU, were able to increase their natural protein intake by a median of 12 g/day when they were challenged with extra protein prior to sapropterin responsiveness testing [15,39]. Two studies observed that small cohorts of adult patients with PKU also tolerated more protein than prescribed by their clinics [41,42].

Creating a predictive mathematical model for natural protein tolerance in PKU would be a particularly valuable tool and help with patient care decision making. Harnessing the data collected in this study, any historical information and new research data for such predictive analytics could give guidance on likely lifelong Phe tolerance according to PKU genotype, age, sex, and treatment course. This is similar to the function of the BIOPKU database [5] that predicts sapropterin response based on variant analysis and is commonly used and highly regarded by the PKU community.

Different systems were used by the PKU centres to allocate Phe intake and that may have influenced the amount of Phe intake prescribed in this study. In accordance with the European PKU guidelines, Centres B, C, E, and H gave fruit and vegetables containing Phe < 76 mg/100 g (except potatoes) in unmeasured amounts, and they attained an overall blood Phe control within target close to 80% or more (except for Centre H with 71% in target range). Many centres using stricter dietary management practices did not achieve higher percentages of blood Phe levels within target range, showing that calculating and measuring the Phe content of all foods does not necessarily improve metabolic control. Various studies [36,43] have previously demonstrated that patients consumed extra Phe (40 and 50%, respectively) when consuming fruit and vegetables containing Phe < 76 mg/100 g without limit but without deterioration in metabolic control [32,33,34].

In our study, the overall mean protein equivalent prescription from protein substitute in patients with PKU from nine centres was 39 g/day (1.1 g/kg/day), which constitutes nearly 66% of mean total protein intake (59 g/day; 1.7 g/kg/day). The optimal protein requirements in PKU are unknown. Compared with the WHO/FAO/UNU 2007 safe levels of protein intake [44], infants and children with PKU in our study were prescribed more than twice the safe level of intake, whilst the mean prescribed total protein intakes of adolescents and adults were approximately 1.2 to 1.5 times the recommended safe intake due to low utilization of protein equivalent from protein substitute. Centres from Northern and Eastern Europe prescribed more protein equivalent from protein substitutes compared with centres from Central and Southern Europe. Similar findings have been reported by Aguiar et al. (2015) [45]. The amount of protein equivalent intake from protein substitutes was affected by the age, severity, and the type of treatment but not by gender. Patients with cPKU were prescribed approximately 1.5 times more protein equivalent from protein substitutes than mPKU patients, and cPKU patients were particularly reliant on protein substitute to provide most of their protein intake, with a protein equivalent from protein substitute to natural protein ratio of 1:0.3 compared with a ratio of 1:0.8 in g/kg/day in mPKU patients. In contrast, patients with HPA had a minimal intake of protein equivalent from protein substitutes, which was usually around 0.1–0.3 g/kg/day.

The total protein intake we report is in line with the recommendations by the European guidelines (140% of FAO/WHO/UNU safe levels of protein intake) [2]; however, 50 patients in our cohort were prescribed less than the safe levels of protein intake. More than 50% of those patients were from Centre A, where access to treatment was limited, particularly to protein substitutes, which may have impacted the amount of total protein prescribed. Another important factor that may impact the interpretation of protein adequacy was that 48% of these patients were overweight. It is suggested in the literature that protein intake must be prescribed according to ideal weight rather than actual weight when considering overweight and obesity. However, the classification of overweight also has its limitations, as patients with a high percentage of muscle mass may have a high BMI [46]. Consideration should be given to measuring body composition (e.g., percentage of lean mass) when determining and recommending protein requirements [47].

The European [2], USA [7] and Australian guidelines [48] advise additional protein substitute (≈120–140% of RDA) to compensate for the inefficiency of protein utilization of amino acids in addition to the pharmacological impact of protein substitute lowering blood Phe levels. They suggest that the same percentage increase in protein equivalent is necessary for both children and adults, and recommendations do not discuss how requirements may change with the severity of PKU. It is expected that patients with mPKU will require less protein equivalent than cPKU, due to a higher natural protein tolerance, therefore there is less concern about inefficient protein utilization. Furthermore, in adulthood, different life stages need protein requirement adjustments, particularly for pregnancy, lactation, and potentially the elderly. There is particular concern that protein requirements may be underestimated in the elderly, who may be at risk of sarcopenia [16,49]. Generally, we have limited data about the protein status and body composition of adults with PKU, particularly if they have had a lifetime of low natural protein intake mainly sourced from plant protein. Further careful examination of total protein intake and requirements is needed.

In this study, all centres used Phe-free L-AA as a source of protein substitute, with four centres prescribing some patients GMP-based protein substitutes to provide all or part of their protein equivalent requirement. In our patient cohort, *n* = 61 adults were taking LNAA from Centres A, E, G, and I that may have contributed to a higher amount of natural protein consumed as they followed a less restricted protein intake. Ahring et al. advocate that LNAA should provide only 25–30% of total daily protein requirements, with the rest of the protein requirements met by natural protein [50]. In general, LNAA are not recommended by the European PKU guidelines due to a lack of efficacy in human studies [2], despite a long history of patient usage. It is suggested that higher amounts of LNAA compete with Phe at the blood–brain barrier, decreasing the uptake of Phe into the brain. Van Vliet et al., in a mice study, found that brain Phe concentrations were similar using LNAA with and without a Phe-restricted diet [51]. More data are needed regarding the quality of dietary intake and neurocognitive outcome when prescribing LNAA.

As reported in several previous studies, we observed that mean Phe intake doubled with BH4-responsive patients treated with sapropterin dihydrochloride. Although the total protein intake did not change, similar to the data reported by Ilgaz et al. [24], the protein equivalent intake from protein substitute decreased. In the systematic review of Ilgaz et al., 51% of patients were able to discontinue protein substitute, compared with only 31% in our study. Overall, our study reported that only 17% of the patient cohort were prescribed sapropterin, partly explained by limited or no access to this treatment by two of our centres. Our results are similar to other studies. In a single centre study, with 2-year follow-up data, Phe intake increased from 421 mg to 1470 mg on sapropterin [52]. The USA PKUDOS registry showed that, after 6 years of sapropterin therapy, Phe tolerance increased from 1000 mg/day to 1539 mg/day while lowering blood Phe levels [53]. Gama et al. have also shown, in a 6-month study, that natural protein intake increased from 11 g/day to 30 g/day in BH4-responsive patients, while maintaining blood Phe levels within range [54]. More long-term Phe tolerance data are required with sapropterin usage.

### Limitations

This study has several limitations. Retrospective dietary intake data were not available for all patients. The results reported dietary intake data prescribed by each centre rather than their actual dietary intakes. Different centres used different methods to report Phe intake. When exchange systems were used, additional Phe intake may be 40–50% higher from low protein foods, but this was not calculated in the Phe allowance. Therefore, some of the data on natural protein tolerance may be underestimated compared with the actual natural protein intake. Adherence to diet was not measured, although the percentage of blood Phe levels within target range was above 65% and, in most centres, between 70 and 88%. Mainly patients with mPKU respond to sapropterin and although these patients have a higher tolerance, natural protein tolerance may already be underestimated in this patient group, as some patients may not have achieved their maximum natural protein tolerance prior to sapropterin use [15]. Patients with LNAA were often given a non-restricted diet, but data were generally not collected on the amount and type of protein consumed. In addition, few centres collected data about the natural protein intake consumed by patients with HPA, with an assumption that they were eating an unrestricted protein intake. Energy intake may also impact blood Phe control, which consequently will impact natural protein tolerance, but these data were not available in our study.

## 5. Conclusions

From these data, some wide-ranging conclusions could be drawn. In general, natural protein intake (g/day) increased during childhood, reaching its highest amount during adolescence. cPKU patients only tolerated 50% of the natural protein of patients with mPKU. Natural protein intake doubled with sapropterin treatment. More data are required to examine protein intake throughout adulthood, including aging. Future PKU guidelines should address protein requirements based on specific characteristics that can impact requirements, e.g., age, PKU severity, body composition accounting for overweight and obesity, and pharmaceutical treatments.

## Figures and Tables

**Table 1:** Dietary practices and frequency of monitoring of the participating centres.

	Centre A	Centre B	Centre C	Centre D	Centre E	Centre F	Centre G	Centre H	Centre I
Number of patients with data on dietary treatment (%)	318 (99)	97 (100)	95 (94)	81 (84)	115 (99)	59 (100)	247 (79)	62 (100)	89 (56)
Dietetic treatment guidelines: Exchange system (Yes/No) -If yes, type of exchange	Yes 1 exchange = 15 mg Phe, 0.5 g protein for fruits = 15 mg Phe, 0.6 g protein = 50 mg Phe, 1 g protein for bread, cereals	Yes 1 exchange = 50 mg Phe = 1 g protein	No	Yes 1 exchange = 20 mg Phe	Yes 1 exchange = 20 mg Phe	No	Yes 1 exchange = 25 mg Phe	No	Yes 1 exchange = 50 mg Phe = 1 g protein
Unmeasured/unlimited use of fruit and vegetables (except potatoes) containing Phe ≤ 75 mg/100 g (Yes/No)	No	Yes	Yes	No	Yes	No	No	Yes	Yes
Foods containing protein ≤0.5 g/100 g given in unlimited amounts	No	Yes	No	No	Yes	No	No	Yes	Yes
Type of protein substitutes used	L-AA (powder, liquid) LNAA (powder, tablets)	L-AA (powder, liquid, tablets) GMP (powder, liquid)	L-AA (powder, liquid, tablets)	L-AA (powder, liquid)	L-AA (powder, liquid, tablets) GMP (powder, liquid, bars) LNAA (powder, tablets)	L-AA (powder, liquid)	L-AA (powder, liquid, tablets, bars) GMP (powder, liquid, bars) LNAA (powder, tablets)	L-AA (powder, liquid)	L-AA (powder, liquid, tablets) GMP (powder, liquid, bars) LNAA (powder, tablets)
Therapeutic target range for blood Phe (μmol/L)	<12 y: 120–360 >12 y: 120–600	<12 y: 120–360 >12 y: 120–600	<12 y: 120–360 >12 y: 120–600	2012–2017: <12 y: 120–600 >12 y: 600–1200 2018: <12 y: 120–360 >12 y: 360–900	<12 y: 120–360 >12 y: 120–480	<12 y: 120–360 >12 y: 120–600	2012–2017: 0–4 y: 180–300 4–8 y: 180–400 8–10 y: 180–600 10–12 y: 180–700 >12 y: 180–900 2018: <12 y: 120–360 >12 y: 360–600	2012–2017: 0–6 y: 120–360 6–9 y: 120–540 10–18 y: 120–600 >18 y: 120–840 2018: <12 y: 120–360 >12 y: 360–600	<12 y: 120–360 >12 y: 120–600
Frequency of clinic visits	0–1 y: monthly; 1–2 y: 2 monthly; 2–3 y: 3 monthly; 4–18 y: 4 times a year >18 y: 6 monthly	0–1 y: 3 monthly; 1–18 y: 6 monthly	0–1 y: 2 monthly; 1–18 y: twice/three times a year; >18 y: once a year	0–1 y: 3 monthly; 1–18 y: 6 monthly; >18 y: once a year	0–6 m: monthly; 6–12 m: 2 monthly; 1–12 y: 3 monthly; 12–18 y: 2/3 times a yearly; >18 y: once/twice a year	0–1 y: monthly; 2–5 y: 2 monthly; 6–12 y: 3/4 monthly; 13–18 y: 6 monthly; >18 y: once a year	0–6 m: 2 monthly; 6–18 m: 3 monthly; 18 m-6 y: 6 monthly; >6 y: once a year	0–1 y: monthly; 1–6 y: 3 monthly; >6 y: 6 monthly	0–1 y: monthly; 1–12 y: 6 monthly; >12 y: once a year
Number of dietitians full time dedicated to IMD	3 (not specific to IMD)	1.3 (specific to PKU)	1.8	5 (not specific to IMD)	2	1	2.7	0	2

Abbreviations: Phe—phenylalanine.; IMD—inherited metabolic disorders; L-AA—amino acid-based protein substitutes; GMP—glycomacropeptide-based protein substitutes; LNAA—large neutral amino acids. Except in the first year of life.

**Table 2:** Mean dietary protein/Phe intakes and metabolic control in each participating centre.

Centre	% Blood Phe Levels in Target	Phe Mean ± SD (Median; Range)		Natural Protein Mean ± SD (Median; Range)		Protein Equivalent from Protein Substitute Mean ± SD (Median; Range)		Total Protein Mean ± SD (Median; Range)	
(N of Patients)	(Mean % ± SD)	mg/day	mg/kg/day	g/day	g/kg/day	g/day	g/kg/day	g/day	g/kg/day
A (*n* = 320)	70 ± 48	658 ± 492 (465; 180–3350)	21 ± 22 (15; 0–176)	13 ± 10 (9; 4–67)	0.5 ± 0.4 (0.4; 0.1–3.5)	17 ± 14 (19; 0–60)	0.7 ± 0.5 (0.7; 0–3.2)	36 ± 11 (35; 7–70)	1.3 ± 0.6 (1.3; 0–4.0)
B * (*n* = 97)	83 ± 30	417 ± 367 (250; 123–1750)	18 ± 19 (12; 0–125)	8 ± 7 (5; 2–35)	0.4 ± 0.4 (0.2; 0–2.5)	52 ± 20 (60; 2–90)	2.0 ± 0.7 (2.0; 0.5–3.5)	61 ± 20 (64; 5–102)	2.3 ± 0.7 (2.4; 0.6–4.5)
C (*n* = 101)	79 ± 53	1065 ± 816 (800; 100–4500)	17 ± 19 (12; 0–173)	21 ± 16 (16; 4–90)	0.4 ± 0.4 (0.3; 0.0–3.5)	41 ± 21 (42; 0–84)	0.8 ± 0.5 (0.8; 0–2.4)	62 ± 20 (65; 10–109)	1.3 ± 0.6 (1.1; 0.4–4.4)
D (*n* = 96)	67 ± 52	887 ± 748 (565; 104–4725)	29 ± 34 (18; 0–219)	22 ± 18 (15; 2–105)	0.8 ± 0.7 (0.5; 0–4.9)	23 ± 21 (22; 0–75)	0.7 ± 0.6 (0.8; 0–2.2)	45 ± 21 (42; 5–108)	1.5 ± 0.6 (1.5; 0.1–4.9)
E + (*n* = 116)	87 ± 49	2097 ± 1738 (1427; 262–9009)	45 ± 37 (32; 0–215)	45 ± 34 (31; 6–182)	1.0 ± 0.8 (0.7; 0.1–4.4)	37 ± 24 (39; 0–94)	0.8 ± 0.5 (0.8; 0–2.4)	80 ± 29 (82; 0–182)	1.7 ± 0.6 (1.6; 0.5–4.4)
F (*n* = 59)	66 ± 32	379 ± 194 (350; 83–1200)	16 ± 12 (12; 3–64)	8 ± 4 (7; 2–24)	0.3 ± 0.2 (0.2; 0.1–1.3)	44 ± 21 (42; 4–90)	1.5 ± 0.5 (1.4; 0.5–3.3)	51 ± 22 (49; 8–110)	1.8 ± 0.5 (1.8; 0.9–3.6)
G (*n* = 314)	65 ± 54	984 ± 951 (579; 0–7500)	18 ± 24 (12; 0–205)	22 ± 20 (14; 0–150)	0.5 ± 0.5 (0.4; 0–4.1)	45 ± 25 (40; 0–120)	1.2 ± 0.7 (1.1; 0–5.3)	67 ± 26 (68; 6–192)	1.7 ± 0.7 (1.6; 0.2–6.4)
H (*n* = 62)	71 ± 44	710 ± 667 (500; 0–3000)	14 ± 18 (7; 0–129)	15 ± 17 (10; 0–60)	0.4 ± 0.7 (0.2; 0–2.6)	53 ± 29 (60; 0–100)	1.2 ± 0.8 (1.2; 0–3.3)	76 ± 19 (84; 16–128)	1.8 ± 0.7 (1.5; 0.7–3.9)
I (*n* = 158)	88 ± 49	942 ± 875 (620; 104–5000)	16 ± 23 (12; 0–171)	21 ± 19 (14; 2–100)	0.5 ± 0.5 (0.4; 0–3.4)	36 ± 23 (36; 0–100)	1.0 ± 0.8 (1.1; 0–3.0)	57 ± 23 (58; 10–120)	1.7 ± 0.7 (1.6; 0.1–3.6)
Total (*n* = 1323)	71 ± 46	904 ± 761 (565; 0–9009)	22 ± 23 (15; 0–219)	19 ± 16 (14; 0–182)	0.5 ± 0.5 (0.4; 0–10.0)	39 ± 22 (40; 0–120)	1.1 ± 0.6 (1.1; 0–5.3)	59 ± 21 (64; 10–192)	1.7 ± 0.6 (1.6; 0.1–6.4)

Abbreviations: *n*—number; SD—standard deviation; Phe—phenylalanine. Data include patients on sapropterin and LNAA. * Paediatric centre only. ^+^ Majority of patients with HPA did not have dietary intake data except for Centre E; 48% of patients from Centre E were mild PKU.

**Table 3:** Dietary protein/Phe intakes presented by gender and age groups.

Sex and Age (Number of Patients)	Mean ± SD (Median; Range)								WHO/FAO/UNU 2007 Safe Levels for Total Protein	% of Mean Total Protein Intake Meeting Recommendations
	Phe		Natural Protein		Protein Equivalent from Protein Substitute		Total Protein			
	mg/day	mg/kg/day	g/day	g/kg/day	g/day	g/kg/day	g/day	g/kg/day	g/kg/day	
Female (*n* = 666)	881 ± 875 (500; 0–5820)	20 ± 25 (13; 0–219)	19 ± 19 (11; 0–181)	0.5 ± 0.6 (0.3; 0.0–4.9)	35 ± 26 (32; 0–120)	1.0 ± 0.7 (1.0; 0.0–4.6)	57 ± 25 (55; 0–167)	1.6 ± 0.7 (1.5; 0.0–5.8)	NA	NA
Male (*n* = 657)	1027 ± 1200 (550; 0–9009)	29 ± 34 (12; 0–215)	22 ± 24 (12; 0–182)	0.5 ± 0.6 (0.4; 0.0–4.2)	35 ± 25 (31; 0–113)	1.0 ± 0.7 (1.0; 0–5.0)	60 ± 28 (56; 0–192)	1.8 ± 0.7 (1.6; 0.1–6.4)	NA	NA
*p*-value	0.218	0.019	0.250	0.019	0.830	0.013	0.850	<0.001		
<2 y (*n*= 47)	318 ± 206 (250; 0–1297)	34 ± 35 (29; 0–162)	7 ± 5 (5; 0–30)	1.0 ± 0.7 (0.9; 0.0–3.5)	7 ± 9 (7; 0–54)	1.1 ± 1.1 (1.1; 0.0–5.3)	17 ± 9 (15; 6–58)	2.4 ± 1.3 (2.4; 0.0–6.4)	1.14 1–1.31 2	210–183%
2–5 y (*n* = 213)	521 ± 468 (352; 100–3802)	29 ± 32 (21; 0–219)	11 ± 10 (8; 0–78)	0.8 ± 0.7 (0.5; 0.0–4.9)	19 ± 15 (19; 0–60)	1.3 ± 0.9 (1.4; 0.0–5.3)	33 ± 12 (32; 0–92)	2.2 ± 0.8 (2.2; 0.0–6.4)	0.94	234%
6–12 y (*n* = 353)	750 ± 756 (450; 0–5360)	22 ± 25 (15; 0–206)	16 ± 17 (10; 0–181)	0.5 ± 0.6 (0.3; 0.0–4.2	36 ± 22 (34; 0–106)	1.1 ± 0.7 (1.1; 0.0–3.3)	54 ± 18 (52; 10–115)	1.7 ± 0.6 (1.7; 0.0–4.2)	0.89 3–0.94 4	191–181%
13–18 y (*n* = 268)	1301 ± 1349 (750; 0–9009)	20 ± 25 (11; 0–215)	27 ± 26 (17; 2–158)	0.5 ± 0.5 (0.3; 0.0–3.5)	47 ± 26 (48; 0–116)	0.8 ± 0.4 (0.8; 0.0–2.3)	75 ± 22 (8; 0–175)	1.3 ± 0.4 (1.2; 0.0–3.6)	0.84 5–0.89 6	155–146%
19–30 y (*n* = 280)	1419 ± 1328 (900; 0–9009)	18 ± 19 (11; 0–123)	31 ± 28 (20; 0–182)	0.4 ± 0.4 (0.3; 0.0–2.5)	48 ± 25 (53; 0–110)	0.7 ± 0.4 (0.8; 0.0–1.6)	78 ± 24 (79; 0–182)	1.2 ± 0.4 (1.2; 0.2–2.5)	0.84	143%
31–40 y (*n* = 107)	1377 ± 1027 (1148; 100–4725)	14 ± 13 (10; 0–62)	30 ± 23 (25; 4–105)	0.4 ± 0.3 (0.3; 0.0–1.8)	47 ± 26 (52; 0–120)	0.6 ± 0.4 (0.7; 0.0–1.3)	78 ± 23 (76; 0–143)	1.0 ± 0.3 (1.0; 0.1–2.0)	0.84	119%
≥41 y (*n* = 55)	1255 ± 917 (750; 350–3750)	7 ± 11 (0; 0–53)	27 ± 20 (15; 7–75)	0.3 ± 0.3 (0.2; 0.0–1.1)	47 ± 24 (50; 0–106)	0.6 ± 0.3 (0.7; 0.0–1.2)	80 ± 22 (77; 35–192)	1.0 ± 0.3 (1.0; 0.4–2.1)	0.84	119%

Abbreviations: *n*—number; Phe—phenylalanine; SD—standard deviation; WHO—World Health Organization; FAO—Food and Agricultural Organization; UNU—United Nations University; NA—not applicable; y—years. ^1^ 7–12 months, ^2^ 0–6 months, ^3^ 11–16 y, ^4^ 1–10 y, ^5^ >16 y, ^6^ 11–16 y.

**Table 4:** Dietary protein/Phe intake data presented in age groups by PKU severity.

PKU Severity	Age Group	Mean ± SD (Median; Range)							
		Phe		Natural Protein		Protein Equivalent from Protein Substitute		Total Protein	
		mg/day	mg/kg/day	g/day	g/kg/day	g/day	g/kg/day	g/day	g/kg/day
HPA	<2 y	656 ± 286 (590; 175–1297)	19 ± 43 (0; 0–162)	14 ± 7 (13; 3–30)	1.3 ± 1.2 (1.1; 0–3.5)	1 ± 3 (0; 0–13)	0.1 ± 0.4 (0; 0–1.7)	18 ± 8 (15; 10–37)	1.4 ± 1.5 (1.7; 0–4.4)
	2–5 y	1385 ± 734 (1359; 150–3802)	26 ± 50 (0; 0–219)	29 ± 15 (28; 4–78)	1.6 ± 1.2 (1.8; 0–4.9)	2 ± 5 (0; 0–36)	0.1 ± 0.3 (0; 0–1.8)	34 ± 13 (31; 5–82)	1.6 ± 1.3 (1.9; 0–4.9)
	6–12 y	2074 ± 1281 (1719; 400–5360)	31 ± 43 (0; 0–206)	44 ± 27 (40; 8–111)	1.3 ± 0.9 (1.2; 0–4.2)	9 ± 13 (0; 0–50)	0.3 ± 0.4 (0; 0–1.6)	58 ± 22 (51; 24–111)	1.6 ± 1.0 (1.6; 0–4.2)
	13–18 y	3325 ± 1577 (3219; 500–7724)	40 ± 41 (34; 0–170)	70 ± 32 (70; 10–158)	1.1 ± 0.8 (1.1; 0–3.5)	13 ± 17 (0; 0–70)	0.2 ± 0.3 (0; 0–1.5)	84 ± 28 (89; 35–158)	1.5 ± 0.7 (1.5; 0–3.6)
	19–30 y	2851 ± 2128 (2300; 239–7473)	24 ± 31 (6; 0–106)	59 ± 44 (50; 7–153)	0.7 ± 0.6 (0.6; 0–2.2)	28 ± 30 (23; 0–108)	0.3 ± 0.4 (0.1; 0–1.1)	85 ± 32 (80; 0–153)	1.2 ± 0.4 (1.1; 0.4–2.2)
	31–40 y	1104 ± 389 (1250; 663–1400)	4 ± 9 (0; 0–22)	23 ± 6 (25; 16–28)	0.3 ± 0.2 (0.3; 0–0.4)	47 ± 3 (45; 45–50)	0.9 ± 0.1 (0.9; 0.8–1.0)	70 ± 4 (70; 66–73)	1.3 ± 0.0 (1.3; 1.2–1.3)
	≥41 y	n/a	n/a	n/a	n/a	n/a	n/a	n/a	n/a
HPA Total		2270 ± 1586 (1910; 150–7724)	29 ± 45 (0; 0–219)	48 ± 33 (41; 3–158)	1.3 ± 1.0 (1.1; 0–4.9)	8 ± 15 (0; 0–108)	0.2 ± 0.4 (0; 0–1.8)	61 ± 31 (52; 0–158)	1.5 ± 1.0 (1.6; 0.0–4.9)
mPKU	<2 y	334 ± 222 (258; 0–1225)	45 ± 37 (41; 0–128)	7 ± 4 (5; 0–25)	1.1 ± 0.7 (1.1; 0–2.6)	7 ± 5 (7; 0–22)	1.3 ± 1.1 (1.3; 0–4.4)	15 ± 5 (15; 6–32)	2.6 ± 1.2 (2.5; 0–6.4)
	2–5 y	615 ± 452 (432; 150–2450)	38 ± 31 (28; 0–175)	13 ± 9 (9; 3–49)	0.9 ± 0.6 (0.7; 0–3.9)	20 ± 14 (20; 0–60)	1.4 ± 0.9 (1.4; 0–4.4)	34 ± 12 (33; 0–72)	2.3 ± 0.8 (2.3; 0–6.4)
	6–12 y	941 ± 664 (750; 184–4306)	28 ± 24 (21; 0–122)	20 ± 14 (16; 4–95)	0.7 ± 0.5 (0.5; 0–2.9)	31 ± 20 (30; 0–84)	1.0 ± 0.7 (1.1; 0–2.8)	53 ± 17 (52; 20–106)	1.7 ± 0.5 (1.7; 0–3.7)
	13–18 y	1637 ± 1131 (1371; 250–6014)	23 ± 21 (16; 0–110)	34 ± 23 (29; 5–125)	0.6 ± 0.4 (0.4; 0–2.2)	37 ± 23 (36; 0–84)	0.6 ± 0.4 (0.6; 0–1.5)	72 ± 23 (74; 0–128)	1.2 ± 0.4 (1.1; 0–2.7)
	19–30 y	1892 ± 1529 (1500; 295–9009)	22 ± 23 (15; 0–123)	40 ± 32 (30; 7–182)	0.6 ± 0.5 (0.5; 0–2.5)	38 ± 22 (38; 0–110)	0.6 ± 0.4 (0.6; 0–1.4)	78 ± 27 (78; 0–182)	1.2 ± 0.4 (1.1; 0.4–2.5)
mPKU	31–40 y	1744 ± 962 (1550; 100–4650)	17 ± 14 (19; 0–50)	38 ± 22 (31; 8–105)	0.5 ± 0.3 (0.4; 0–1.8)	41 ± 24 (48; 0–100)	0.5 ± 0.3 (0.6; 0–1.2)	80 ± 20 (78; 10–128)	1.1 ± 0.3 (1.1; 0.1–2.0)
	≥41 y	1433 ± 995 (1057; 363–3750)	8 ± 14 (0; 0–53)	33 ± 23 (24; 7–75)	0.4 ± 0.3 (0.3; 0–1.1)	42 ± 24 (42; 0–76)	0.5 ± 0.4 (0.4; 0–1.1)	77 ± 16 (78; 35–105)	1.0 ± 0.3 (0.9; 0.4–1.5)
mPKU Total		1262 ± 1166 (900; 0–9009)	28 ± 26 (21; 0–175)	26 ± 23 (20; 0–182)	0.7 ± 0.5 (0.5; 0–3.9)	32 ± 22 (30; 0–110)	0.9 ± 0.7 (0.9; 0–4.4)	60 ± 28 (58; 0–182)	1.7 ± 0.7 (1.5; 0–6.4)
cPKU	<2 y	251 ± 102 (234; 83–500)	38 ± 23 (34; 0–125)	5 ± 2 (5; 0–10)	0.8 ± 0.4 (0.8; 0–2.5)	12 ± 9 (9; 2–54)	1.8 ± 0.9 (1.5; 0–5.3)	18 ± 10 (14; 6–58)	2.6±1.1 (2.5; 0–6.3)
	2–5 y	337 ± 155 (310; 100–1631)	24 ± 13 (22; 0–88)	7 ± 3 (7; 0–35)	0.5 ± 0.2 (0.5; 0–1.8)	26 ± 11 (24; 2–60)	1.8 ± 0.6 (1.7; 0–5.3)	33 ± 11 (31; 6–92)	2.3±0.6 (2.2; 0.7–6.3)
	6–12 y	414 ± 207 (400; 0–2000)	14 ± 8 (13; 0–70)	9 ± 8 (8; 0–75)	0.3 ± 0.4 (0.3; 0–4.2)	45 ± 19 (42; 0–106)	1.4 ± 0.5 (1.4; 0–3.3)	54 ± 18 (51; 23–115)	1.7±0.5 (1.7; 0.6–3.5)
	13–18 y	654 ± 474 (500; 0–4500)	11 ± 9 (9; 0–66)	14 ± 11 (11; 2–90)	0.2 ± 0.2 (0.2; 0–1.3)	58 ± 22 (60; 0–116)	1.0 ± 0.4 (1.0; 0–2.3)	72 ± 20 (74; 16–175)	1.2±0.3 (1.2; 0.3–2.4)
	19–30 y	1033 ± 904 (719; 0–6096)	13 ± 14 (10; 0–87)	23 ± 20 (15; 0–129)	0.3 ± 0.3 (0.2; 0–1.8)	53 ± 24 (60; 0–106)	0.8 ± 0.4 (0.8; 0–1.6)	76 ± 21 (78; 0–146)	1.2±0.3 (1.2; 0.2–2.4)
	31–40 y	1206 ± 1060 (845; 171–4725)	12 ± 13 (12; 0–62)	26 ± 23 (17; 4–105)	0.3 ± 0.3 (0.2; 0–1.3)	50 ± 26 (60; 0–120)	0.6 ± 0.4 (0.7; 0–1.3)	78 ± 25 (77; 0–143)	1.0±0.3 (1.0; 0.1–1.9)
	≥41 y	1284 ± 949 (714; 350–3085)	6 ± 11 (0; 0–45)	27 ± 20 (17; 7–71)	0.3 ± 0.3 (0.2; 0–1.0)	48 ± 25 (54; 16–106)	0.6 ± 0.3 (0.7; 0–1.2)	83 ± 25 (77; 45–192)	1.0±0.3 (1.0; 0.4–2.1)
cPKU Total		593 ± 585 (425; 0–6096)	16 ± 13 (13; 0–125)	13 ± 14 (9; 0–129)	0.4 ± 0.3 (0.3; 0–4.2)	44 ± 23 (40; 0–120)	1.2 ± 1.6 (1.2; 0–5.3)	57 ± 25 (54; 0–192)	1.7 ± 0.7 (1.5; 0–6.3)

Abbreviations: SD—standard deviation; y—years; Phe—phenylalanine; HPA—hyperphenylalaninemia; mPKU—mild PKU; cPKU—classical PKU.

**Table 5:** Differences of mean protein/Phe values between PKU severities.

Age Group	Differences by Severities	Difference of Mean Values between Severities					
		Phe		Natural Protein		Protein Equivalent from Protein Substitute	
		mg/Day	mg/kg/Day	g/Day	g/kg/Day	g/Day	g/kg/Day
<2 y	*HPA-cPKU* *mPKU-cPKU*	+405 +83	−19 +7	+9 +2	+0.5 +0.3	−12 −5	−1.7 −0.5
2–5 y	*HPA-cPKU* *mPKU-cPKU*	+1048 +278	+2 +14	+22 +6	+1.1 +0.4	−24 −6	−1.7 −0.4
6–12 y	*HPA-cPKU* *mPKU-cPKU*	+1660 +527	+17 +14	+35 +11	+1.0 +0.4	−36 −14	−1.1 −0.4
13–18 y	*HPA-cPKU* *mPKU-cPKU*	+2671 +983	+29 +12	+56 +20	+0.9 +0.4	−45 −21	−0.8 −0.4
19–30 y	*HPA-cPKU* *mPKU-cPKU*	+1818 +859	+11 +9	+36 +17	+0.4 +0.3	−25 −15	−0.5 −0.2
31–40 y	*HPA-cPKU* *mPKU-cPKU*	−102 +538	−8 +5	−3 +12	0 +0.2	−3 −9	+0.3 −0.1
≥41 y	*HPA-cPKU* *mPKU-cPKU*	n/a +149	n/a +2	n/a +6	n/a +0.1	n/a −6	n/a −0.1

Abbreviations: y—years; Phe—phenylalanine; HPA—hyperphenylalaninemia; mPKU—mild PKU; cPKU—classical PKU; n/a—not available. Total protein intakes are not shown in the table as they were similar between different severities.

**Table 6:** Dietary protein/Phe intake data comparing patients on diet-only treatment vs. sapropterin (BH4).

Type of Treatment (Number of Patients)	Mean ± SD (Median; Range)							
	Phe		Natural Protein		Protein Equivalent from Protein Substitute		Total Protein	
	mg/Day	mg/kg/Day	g/Day	g/kg/Day	g/Day	g/kg/Day	g/Day	g/kg/Day
BH4 (*n* = 222)	1649 ± 919 (1500; 100–6774)	50 ± 43 (24; 0–173)	35 ± 19 (30; 5–139)	0.9 ± 0.6 (0.7; 0.0–3.5)	24 ± 22 (22; 0–84)	0.6 ± 0.5 (0.5; 0–2.8)	63 ± 23 (62; 10–139)	1.5 ± 0.6 (1.4; 0.1–4.5)
Diet-only (*n* = 1101)	846 ± 1036 (487; 0–9009)	29 ± 30 (32; 6–192)	19 ± 83 (10; 0–182)	0.5 ± 1.5 (0.4; 0–4.9)	37 ± 25 (34; 0–120)	1.2 ± 0.7 (1.1; 0–5.3)	59 ± 85 (54; 0–192)	1.7 ± 1.6 (1.6; 0–6.4)
*p*-value	<0.001	<0.001	<0.001	<0.001	<0.001	<0.001	0.067	<0.001

Abbreviations: *n*—number; SD—standard deviation; Phe—phenylalanine; BH4—sapropterin.

## Data Availability

Data are contained within the article.

## Supplementary Materials

The following supporting information can be downloaded at: https://www.mdpi.com/article/10.3390/nu16172909/s1, Table S1: Number of patients with each PKU severity by centre.
