# Supplementary material for: Longitudinal Dietary Intake Data in Patients with Phenylketonuria from Europe: The Impact of Age and Phenylketonuria Severity
Source: Nutrients. 2024 Aug 31;16(17):2909. doi: 10.3390/nu16172909 (PMC11396810; doi:10.3390/nu16172909)
Supplement: Supplementary file 1 [file nutrients-16-02909-s001.zip › nutrients-3147655-supplementary/nutrients-3147655-supplementary.pdf]

Supplementary Table S1. Number of patients with each PKU severity by centre.

	**Centre A**	**Centre B**	**Centre C**	**Centre D**	**Centre E**	**Centre F**	**Centre G**	**Centre H**	**Centre I**
**HPA (number of patients; % of total number of patients in that centre)**	97; 30%	NA	17; 16%	19; 20%	27; 23%	6; 10%	68; 22%	10; 16%	81; 51%
**Mild PKU (number of patients; % of total number of patients in that centre)**	69; 22%	38; 39%	46; 46%	21; 22%	54; 47%	8; 14%	82; 26%	13; 21%	26; 16%
**Classical PKU (number of patients; % of total number of patients in that centre)**	153; 48%	56; 58%	33; 33%	56; 58%	32; 28%	45; 76%	160; 51%	39; 63%	51; 32%
**Unknown (number of patients; % of total number of patients in that centre)**	1; 0.3%	3; 3%	5; 5%	0; 0%	3; 3%	0; 0%	4; 1%	0; 0%	0; 0%
**Total number patients**	320	97	101	96	116	59	314	62	158

Abbreviations: PKU, Phenylketonuria; HPA, Hyperphenylalaninemia; NA, not applicable. Severity of patients was defined by variants or diagnostic Phenylalanine levels depending availability.
